# Influence of Bacterial Competitors on *Salmonella enterica* and Enterohemorrhagic *Escherichia coli* Growth in Microbiological Media and Attachment to Vegetable Seeds

**DOI:** 10.3390/foods10020285

**Published:** 2021-01-31

**Authors:** Da Liu, Ronald Walcott, Kevin Mis Solval, Jinru Chen

**Affiliations:** 1 Department of Food Science and Technology, The University of Georgia, Griffin, GA 30602, USA; dliu@uga.edu (D.L.); kmissolval@uga.edu (K.M.S.); 2 Department of Plant Pathology, The University of Georgia, Athens, GA 30602, USA; rwalcott@uga.edu

**Keywords:** *Salmonella*, EHEC, vegetable seeds, biological control, bacterial attachment, growth inhibition

## Abstract

Interests in using biological agents for control of human pathogens on vegetable seeds are rising. This study evaluated whether probiotic bacterium *Lactobacillus rhamnosus* GG, bacterial strains previously used as biocontrol agents in plant science, as well as a selected plant pathogen could compete with foodborne human pathogens, such as *Salmonella enterica* and enterohemorrhagic *Escherichia coli* (EHEC), for growth in microbiological media and attachment to vegetable seeds; and to determine whether the metabolites in cell-free supernatants of competitive bacterial spent cultures could inhibit the growth of the two pathogens. The results suggest that the co-presence of competitive bacteria, especially *L. rhamnosus* GG, significantly (*p* < 0.05) inhibited the growth of *Salmonella* and EHEC. Cell-free supernatants of *L. rhamnosus* GG cultures significantly reduced the pathogen populations in microbiological media. Although not as effective as *L. rhamnosus* GG in inhibiting the growth of *Salmonella* and EHEC, the biocontrol agents were more effective in competing for attachment to vegetable seeds. The study observed the inhibition of human bacterial pathogens by competitive bacteria or their metabolites and the competitive attachment to sprout seeds among all bacteria involved. The results will help strategize interventions to produce vegetable seeds and seed sprouts free of foodborne pathogens.

## 1. Introduction

Consumption of raw and lightly cooked sprouts have been linked to multiple high-profile outbreaks of human gastrointestinal infections [1,2,3]. The most likely source of pathogens in sprout-associated outbreaks is contaminated seeds [4]. Pathogens such as *Salmonella* and *E. coli* O157:H7, when present on seeds, can grow rapidly from a low contamination level of ca. 0.1 log CFU/g to as high as 10^6^ log units under sprouting conditions [5]. Although seeds are not generally regarded as a contamination source of fresh produce other than sprouts, recent research by Deering et al. [6] reported the presence of *E. coli* O157:H7 in mature tomato fruits grown from seeds contaminated with the pathogen. Therefore, the sanitary condition of seeds needs to be addressed in a great effort to reduce the growing incidence of foodborne outbreaks associated with fresh produce.

Hypochlorite-based sanitizers have been recommended by the U.S. Food and Drug Administration to inactivate *Salmonella* and enterohemorrhagic *Escherichia coli* (EHEC) on vegetable seeds at the postharvest stage [7,8]. In general, a 3-log population reduction can be reached by applying the sanitizer on sprout seeds for 10 min [9]. However, these pathogens cannot be reliably eliminated due to bacterial strain-specific resistance to chlorine and/or the internalization of pathogen cells into the protected niches of vegetable seeds [10,11].

The potential of using antagonistic microorganisms as control agents on harvested vegetable seeds has been discussed [12,13]. Inoculation of *Acidovorax avenae* subsp. *Avenae* on watermelon seeds before planting could reduce the transmission of bacterial fruit blotch of cucurbits caused by *Acidovorax avenae* subsp. *citrulli* by 96.5% [14]. Treating alfalfa seeds with *Pseudomonas* strain 2-79 has been reported to reduce the population of *Salmonella* by 1-2 log units during sprouting [15]. An advantage of using biocontrol agents over chemical/physical disinfection for pathogen control is that once the microorganisms used as biocontrol agents establish a population on vegetable seeds/plants, sustainable protection can be obtained throughout the sprouting/cultivation process [16].

The mechanisms of utilizing biocontrol agents to improve the microbial safety of vegetable seeds include growth competition, via accessing available nutrients between plant/human pathogens and the microorganisms used as biocontrol agents, and the inhibitory effects of antagonistic metabolites, produced by biocontrol agents towards plant/human pathogens [17]. For instance, the preemptive colonization of orange flowers by *Pseudomonas fluorescens* A506 can effectively prevent future *Erwinia amylovora* infections [18]. Active production of lactic acid and bacteriocin by *Lactobacillus* spp. has led to its application as *Salmonella*-control agents on meat products [19]. Beyond that, metabolites such as fatty acids and biosurfactants produced by *Bacillus* spp. have been reported to reduce adhesion and biofilm formation by human pathogen cells on food contact surfaces [20].

The objectives of this study were to observe whether a probiotic bacterium, strains of bacteria previously used as biocontrol agents in plant science, as well as a selected plant pathogen could compete with foodborne human pathogens such as *Salmonella* and EHEC for growth in microbiological media and attachment to vegetable seeds, and to determine whether the metabolites in cell-free supernatants of spent cultures of competitive bacteria could significantly inhibit the growth of *Salmonella* and EHEC under laboratory conditions.

## 2. Materials and Methods 

### 2.1. Bacterial Strains and Vegetable Seeds

Four *S. enterica*, three *E. coli* O157:H7, and one *E. coli* O104:H4 strain were used in the study due to their previous association with sprout- or fresh produce-associated outbreaks of infections (Table 1). Cells of each pathogen strain resistant to nalidixic acid (NA) were selected on tryptic soy agar (TSA) containing 50 μg/mL of NA (NATSA). The NA resistant derivates were confirmed in a comparative preliminary study as appropriate surrogates of the wildtype parental strains. Three bacterial strains previously used as biological control agents including *P. fluorescens* A506, *Bacillus mojavensis* RRC 101, and *B. subtilis* ATCC 6051 were from the culture collection of Dr. Ronald Walcott (Table 1). A well-characterized probiotic strain, *Lactobacillus rhamnosus* GG was obtained from a commercial source (Culturelle^®^, i-Health Inc., Cromwell, CT, USA). A plant pathogen, *Pseudomonas syringae* pv. *Tomato* DC3000 (Pst DC3000), was also included to observe its possible interactions with the human pathogenic bacterial strains used in the study. All bacterial cultures were maintained at –80 °C until use. The microbiological media used were from Becton, Dickinson, and Company (Sparks, MD, USA) unless specified.

Seeds of alfalfa (*Medicago sativa*), fenugreek (*Trigonella foenum-graecum*), lettuce (*Lactuca sativa* ‘Iceberg’), and tomato (*Solanum lycopersicum* ‘Roma’) were included in the study. These seeds or the fresh produce developed from them had a previous link to the outbreaks of human gastrointestinal infections. The seeds were purchased from Twilley Seed Company (Hodges, SC, USA) and stored at 10 °C until use.

### 2.2. Competitive Growth between *Salmonella* or EHEC and Selected Plant Pathogen, Probiotic Strain, or Biocontrol Agents in Microbiological Media

A previously described [21], 1:1 (*v*/*v*) mixture of De Man, Rogosa and Sharpe (MRS) broth and tryptic soy broth (TSB) (M/T broth) was used in this portion of the study to minimize the influence of microbiological media on the growth of *L. rhamnosus* GG *vs*. other bacterial strains. Fresh M/T broth was prepared by aseptically mixing an equal volume of pre-autoclaved MRS broth and TSB before use. Individual cultures of *Salmonella* and EHEC strains were grown at 37 °C and those of the biocontrol agents/plant pathogen at 25 °C, all in 10 mL M/T broth for 18 h. 

The competitive growth study was performed according to a previously described protocol [21] with modifications. A *Salmonella* or EHEC overnight culture (1.0 mL), pre-diluted to ca. 3.0 log CFU/mL with M/T broth, was mixed, respectively with 1.0 mL of the overnight culture of *P. fluorescens* A506, *B. mojavensis* RRC 101, *B. subtilis* ATCC 6051, *L. rhamnosus* GG, and their cocktails, as well as plant pathogen Pst DC3000, all pre-diluted to ca. 5.0 log CFU/mL with M/T broth. One of the cocktails (Cocktail 1) had an equal number of *B. subtilis* ATCC6051, *B. mojavensis* RRC101, and *P. fluorescens* A506 cells; whereas the other cocktail (Cocktail 2) contained an equal population of the same three bacterial strains plus *L. rhamnosus* GG. The cocktails were vortexed vigorously to ensure uniformity. The mixed cultures of human pathogens and their competitors were incubated at 25 °C with agitation at 100 rpm on a platform shaker (Orbit Shaker, Lab-Line Instruments, Inc., Lumberton, NC, USA). A separate set of tissue culture plates holding the mixture of *L. rhamnousus* GG and each *Salmonella*/*E. coli* strain was prepared in the same manner except that the incubation temperature was set to 37 °C since *L. rhamnousus* GG, unlike other competitive bacterial strains used in the study, has optimal growth at this temperature. The control samples were prepared by mixing 1 mL overnight culture of each *S. enterica* and EHEC strain pre-diluted to ca. 3 log CFU/mL in M/T broth with 1 mL of M/T broth for incubation at both 37 and 25 °C. Broth culture samples were collected after 6, 12, 24, 48, and 72 h of the incubation at the set temperature, and the collected samples were diluted in phosphate-buffered saline (PBS; pH 7.4), and appropriate dilutions were plated in duplicate on XLT4 or sorbitol MacConkey agar supplemented with 50 μg/mL NA (NASMAC). Colonies of *S. enterica*, *E. coli* O157:H7, and *E. coli* O104:H4 were counted after 24–48 h of incubation at 37 °C. 

### 2.3. Competitive Attachment between *Salmonella* or EHEC and Selected Plant Pathogen, Probiotic Strain, or Biocontrol Agents to Vegetable Seeds 

Competitive attachment between *Salmonella* or EHEC cells and those of selected plant pathogen, probiotic strain, and biocontrol agents to vegetable seeds was studied using a previously published protocol [22] with modifications. Two grams of each type of vegetable seeds described above were placed in 50-mL centrifuge tubes (Fisher Scientific, Asheville, NC, USA) and sanitized with 10 mL of 20,000 ppm sodium hypochlorite solution (pH 6.8; BD) at room temperature for 10 min with gentle mixing. The sanitizer solution was then decanted, and residual chlorine was removed by soaking the seeds in 10 mL Dey-Engley neutralizing broth (BD) for 10 min and rinsing twice, each with 10 mL of sterilized deionized water. 

*Salmonella* or EHEC cultures were grown in M/T broth at 37 °C, while individual competitive bacterial cultures were grown in the same broth at 25 °C till the cell concentrations of each culture reached ca. 10^9^ CFU/mL. An equal volume of the four *Salmonella* or EHEC cultures were mixed and then diluted in PBS to create two 4-strain pathogen cocktails having a cell population of ca. 10^4^ CFU/mL. Individual or mixed competitive bacterial cultures were also diluted to 10^4^ CFU/mL in PBS. All samples were mixed vigorously using a vortex device to ascertain that cells were evenly distributed.

Ten milliliters of the *Salmonella* or EHEC cocktail was mixed with an equal volume of each competitive strain/mixture in Falcon centrifuge tubes containing sanitized vegetable seeds. Ten milliliters of each *Salmonella* or EHEC cocktail and 10 mL of PBS were added to a separate set of seeds as controls. The precise inoculation levels were determined by plating 0.1 mL of appropriately diluted cell suspensions on TSA or NATSA. Vegetable seeds in the centrifuge tubes were agitated horizontally at 100 rpm in an orbital platform shaker at 20 °C for 5 h. The inoculums were then decanted, and the seeds were rinsed twice, each with 10 mL sterile water for 1 min with gentle mixing. Seeds were then soaked overnight at 4 °C in 5 mL of PBS to release attached bacterial cells. On the next day, seed samples were vortexed at maximal speed (3200 rpm) (Fisher Scientific) for 50 s before 0.1 mL of soaking solution was spread plated on XLT4 or NASMAC plates in duplicate. The plates were incubated at 37 °C for 24–48 h for the enumeration of *Salmonella* and *E. coli* colonies. The percentage of attached cells in the total number of cells used in the experiment was used to express the ability of each pathogen cocktail in attaching to the vegetable seeds as affected by the presence of bacterial competitors. 

### 2.4. Effect of Metabolites in Cell-Free Supernatants (CFS) of the Spend Cultures of Biocontrol Agents and Probiotic Strain on *Salmonella* and EHEC 

For the preparation of cell-free supernatants, *L. rhamnosus* GG was grown in 10 mL MRS broth at 37 °C for 72 h. Each biological control agent was grown individually in 10 mL TSB at 25 °C for 72 h. Obtained bacterial cultures were centrifuged at 6,000× *g* for 10 min. The resulting supernatants of the bacterial cultures were carefully removed. Suspending bacterial cells in the supernatants were removed by filtration using 0.45-μm sterile syringe filters (Fischer Scientific, Hampton, NH, USA). A one-milliliter aliquot of each filtered supernatant was mixed with 1 mL of an overnight culture of each *Salmonella* and *E. coli* strain, pre-diluted to ca. 10^5^ CFU/mL with PBS. The pathogens cells in the supernatants of competitive bacterial spent cultures were incubated at 25 °C, and samples were collected after 2, 4, 8, 12, 24, and 48 h during incubation. Collected samples (0.1 mL) were plated in duplicate on XLT4 or NCSMAC agar plates, and colonies of *Salmonella* and EHEC were counted after incubation at 37 °C for 24–48 h. 

### 2.5. Statistical Analysis

Every sample in the experiment has a duplicate, and each experiment was conducted in triplicates. The mean populations of different *Salmonella* and EHEC strains as affected by (1) the presence of different competitive agents or their cell-free metabolites, (2) different pathogen strains used, and (3) sampling times were arranged into the general linear model of the SAS software (Version 4, SAS Institute, Cary, NC, USA). Fisher’s Least Significance Difference test was used to separate the means. The same statistical protocol was also used to compare the percentage of attachment of *Salmonella* and EHEC cells to alfalfa, fenugreek, lettuce, and tomato seeds. Type III error test was performed to determine the significance of each variable and interaction between or among different variables. For all comparisons, *p*-value less than 0.05 was considered significant. 

## 3. Results

### 3.1. Competitive Growth between *Salmonella*/EHEC and Bacterial Competitors

Overall mean populations of all four *Salmonella* or EHEC strains in co-cultures with individual competitive bacterial strains/cocktails are shown in Table 2. The mean populations of both *Salmonella* and EHEC in the co-cultures were significantly lower (*p* < 0.05) than those in the control samples. However, the margins of differences were sometimes small; *Salmonella* and EHEC population differences in co-cultures with competitive bacterial strains other than *L. rhamnousus* GG were all below 1 log unit. Among the three tested biocontrol agents, *Salmonella* and EHEC populations in co-culture with *P. fluorescens* A506 were the lowest. EHEC or *Salmonella* populations in co-culturing with *B. mojavensis* RRC 101 were similar to those with *B. subtilis* ATCC 6051. The presence of plant-pathogen, Pst DC3000 in co-cultures also slightly retarded the growth of *Salmonella* and EHEC during the 72-h co-incubation period.

The *Salmonella* populations in the co-culture with *L. rhamnousus* GG were significantly lower than those in other co-cultures, at both 37 °C and 25 °C (Table 2). The mean population of *Salmonella* in co-culture with *L. rhamnousus* GG at 37 °C was 5.3 log units lower than the population in the control and 1.8 log lower than the *Salmonella* population in the co-culture grown at 25 °C. Adding *L. rhamnousus* GG to Cocktail 1 did not result in a greater (*p* > 0.05) impact on *Salmonella* growth, but a different result was observed with EHEC over the 72-h co-incubation period. Mean *Salmonella* and EHEC populations in the co-culturing with Cocktail 2 were significantly higher than those with *L. rhamnousus* GG at both 25 °C and 37 °C. The populations of EHEC were significantly lower when they were co-cultured with Cocktail 2 compared to the co-cultures with individual competitive bacterial strains. Mean *Salmonella* populations were similar in the co-cultures with the two cocktails and *P. fluorescens* A506, and the three populations were significantly lower than those in the co-cultures with *B. mojavensis* RRC 101, *B. subtilis* ATCC 6051, and Pst DC3000. 

When co-cultured with their growth competitors, the mean populations of *S*. Cubana and *S*. Stanley were significantly lower (*p* < 0.05) than those of *S*. Montevideo and *S*. Baildon (Table 2). *E. coli* H1730 and K4492 populations were significantly higher than the mean populations of *E. coli* F4546 and BAA 2326. A significant increase in the mean populations of *Salmonella* and EHEC was seen at most sampling points except the *Salmonella* population at the 24 and 48 h sampling points. 

Different from the results of overall statistical analysis shown in Table 2, no significant difference was observed among the populations of the four individual *Salmonella* strains recovered from the co-cultures with different bacterial competitors at 25 °C (Table 3), except co-incubation with *L. rhamnosus* GG. Furthermore, all four *Salmonella* strains had similar mean cell populations in the co-cultures with the same growth competitors. Different observations were made with EHEC, and co-culture with *L. rhamnosus* GG only significantly reduced the population of H1730 and K4492 (Table 4). The mean populations of *E. coli* BAA2326 were the lowest among all EHEC cultures.

**Figure 1:**
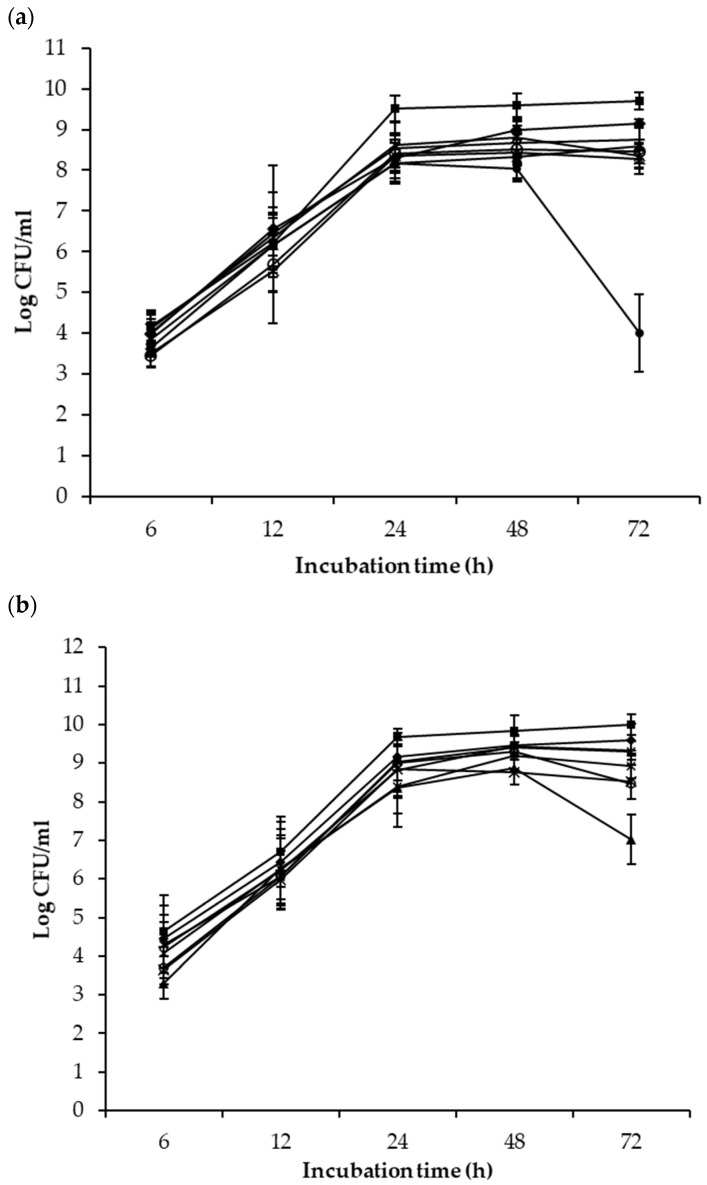
Mean populations of all four strains of *S. enterica* (**a**) or *E. coli* (**b**) in the controls (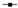) and the mixed cultures with *P. fluorescenes* A506 (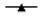), *B. subtilis* ATCC 6051 (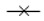), *B. mojavensis* RRC 101 (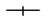), Cocktail 1 (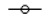), Cocktail 2 (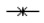), *L. rhamnousus* GG (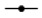) and *P. syringae* pv. *tomato* DC3000 (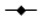) after 6, 12, 24, 48 and 72 h of co-incubation at 25 °C.

According to the results in Figure 1a, the mean populations of *Salmonella* in the control and co-cultures with each competitive bacterium increased exponentially within the first 24 h of incubation at 25 °C before entering the stationary growth phase. The population difference between the controls and co-cultures with individual bacterial strains was from 0.9 to 1.3 log units at the 24 h sampling point. No significant improvement in *Salmonella* inhibition was observed at the 48-h sampling point. At the 72-h sampling point, however, the mean *Salmonella* population in the co-culture with *L. rhamnosus* GG was 4.5 log units lower than the control. *Salmonella* population differences among other co-cultures ranged from 0.5 to 1.4 log units at this sampling point.

Compared to the control, the mean populations of all four tested EHEC strains were 0.7–2.9 log units lower when co-incubated with the bacterial competitors throughout the 72-h incubation period at 25 °C (Figure 1b). At the 72-h sampling point, the EHEC population was 1.4 log CFU/mL lower in the co-cultures with Cocktail 1, 1.3 log CFU/mL lower in co-culture with Cocktail 2, and 2.8 log CFU/mL lower in co-culture with *L. rhamnosus* GG than the population in the control culture. Similar to what was observed with *Salmonella*, a significant decrease in the EHEC population was observed between 48 and 72 h in co-culture with *L. rhamnosus* GG, but the level of population decrease was less profound compared to that of *Salmonella* (Figure 1a).

**Figure 2:**
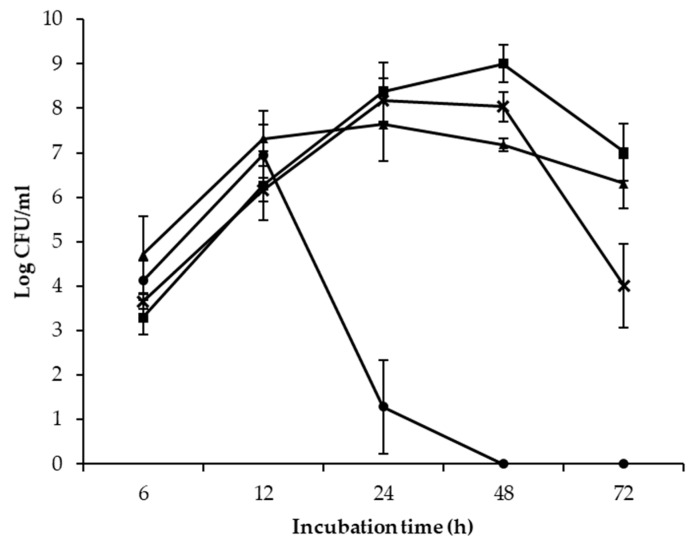
Mean population s of all four *S. enterica* and *E. coli* when co-cultured with *L. rhamnousus* GG at 37 °C (*S. enterica*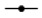; *E. coli*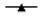) and 25 °C (*S. enterica*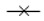; *E. coli*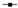) after 6, 12, 24, 48, and 72 h of incubation.

The competitiveness of *L. rhamnosus* GG towards *Salmonella* and EHEC growth was much stronger at 37 °C compared to 25 °C (Figure 2). After 24 h of co-incubation with *L. rhamnosus* GG, the mean populations of all four tested *Salmonella* or EHEC strains were lower at 37 °C than at 25 °C. No *Salmonella* cells were detected from the co-culture with *L. rhamnosus* GG at 37 °C after the 48-h sampling point (detection limit: 10 CFU/mL). The mean populations of EHEC recovered from the co-cultures with *L. rhamnosus* GG were relatively higher than those of *Salmonella* incubated at the same temperatures. 

### 3.2. Competitive Attachment to Vegetable Seeds by *Salmonella*/EHEC as Affected by Bacterial Competitors

The mean percentages of attached cells of *Salmonella* or EHEC cocktail to the four types of vegetable seeds were significantly lower (*p* < 0.05) when the competitive bacterial strains were present (Table 5). When no competitive bacterial strains were used, the mean percentage of attachment of *Salmonella* and EHEC cells were 10.5% and 3.9%, respectively. In the co-cultures with *P. fluorescens* A506, both *Salmonella* and EHEC strains had the lowest percentages of attachment, 7 and 2.4%, respectively, on the vegetable seeds. The percentages of attachment of *Salmonella* in the co-cultures with *B. mojavensis* RRC 101 (7.9%), *B. subtilis* ATCC 6051 (8.1%), and Pst DC 3000 (8.2%) were statistically similar (*p* > 0.05) but were significantly lower than the percentage of attachment in co-cultures with *L. rhamnosus* GG (9.4%) and the competing cocktail (9.0%). Except for the controls, the highest percentages of attachment of EHEC were seen in the co-cultures with *L. rhamnosus* GG (3.4%) and Pst DC3000 (3.5%), followed by the bacterial cocktail (3.1%), *B. mojavensis* RRC 101 (2.9%), and *B. subtilis* ATCC 6051 (2.8%). The percentages of attachment of both *Salmonella* and EHEC cells were the highest on fenugreek seeds (12.5% and 6.7%), followed by alfalfa (11.8% and 2.0 %) and lettuce (8.9% and 1.7%) seeds. No attachment was detectable from tomato seeds used in the study.

### 3.3. Inhibition of *Salmonella* and EHEC by the CFS of *L. rhamnousus* GG Spent Cultures

**Figure 3:**
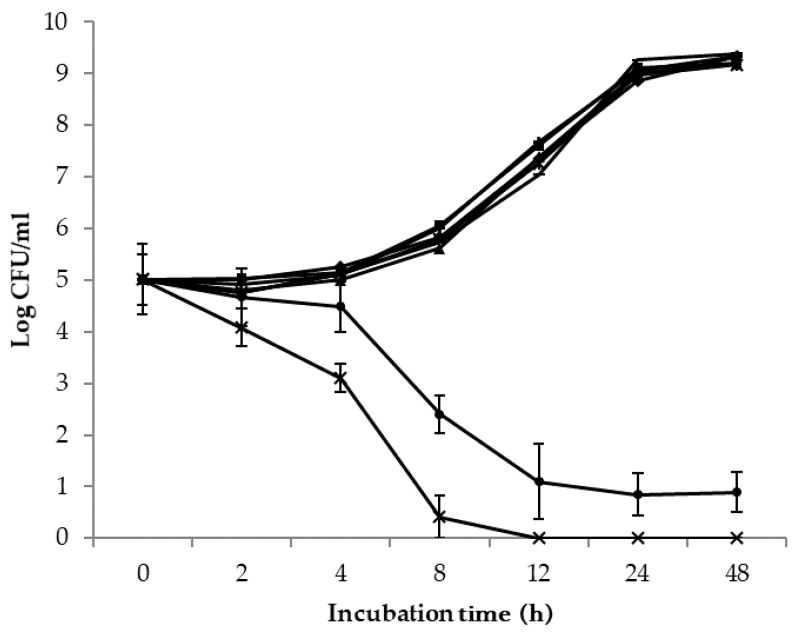
Mean populations of all four *S. enterica* and EHEC cultured in PSB amended with the cell-free supernatants of *P. fluorescenes* A506 (*S. enterica*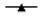; EHEC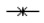), *B. subtilis* ATCC 6051 (*S. enterica*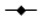; EHEC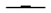), *B. mojavensis* RRC 101 (*S. enterica*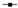; EHEC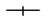) and *L. rhamnousus* GG (*S. enterica*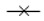; EHEC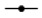) at the 2, 4, 8, 12, 24 and 48 h sampling points.

Changes in the mean population of all four *Salmonella* and EHEC strains grown in PBS amended with the CFS of 72-h spent cultures of the three individual biocontrol agents and *L. rhamnousus* GG are shown in Figure 3. The addition of *L. rhamnousus* GG CFS to the diluted *Salmonella* and EHEC cultures resulted in significant (*p* < 0.05) reductions in the populations of *Salmonella* and EHEC. The mean populations of the four *Salmonella* strains reduced approximately 1 log unit after 2 h of incubation. *Salmonella* cells became undetectable (<10 CFU/mL) at the 12 h sampling point and forward. At the 24 h sampling point, a total of 5 log unit reduction was achieved. During the incubation period from the 4 h to 6 h sampling point, the mean population of the four EHEC strains decreased by 2.4 log CFU/mL. A total of 4 log CFU/mL reduction was observed after the 24 h incubation period. In comparison, the growth of *Salmonella* and EHEC in other co-cultures was not inhibited and the pathogen populations increased exponentially before entering the stationary phase after 24 h of incubation. The populations of *Salmonella* and EHEC recovered from these co-cultures did not differ from each other throughout the incubation period, with the maximum population difference of 0.6 log units for *Salmonella* and 0.4 log units for EHEC. 

**Figure 4:**
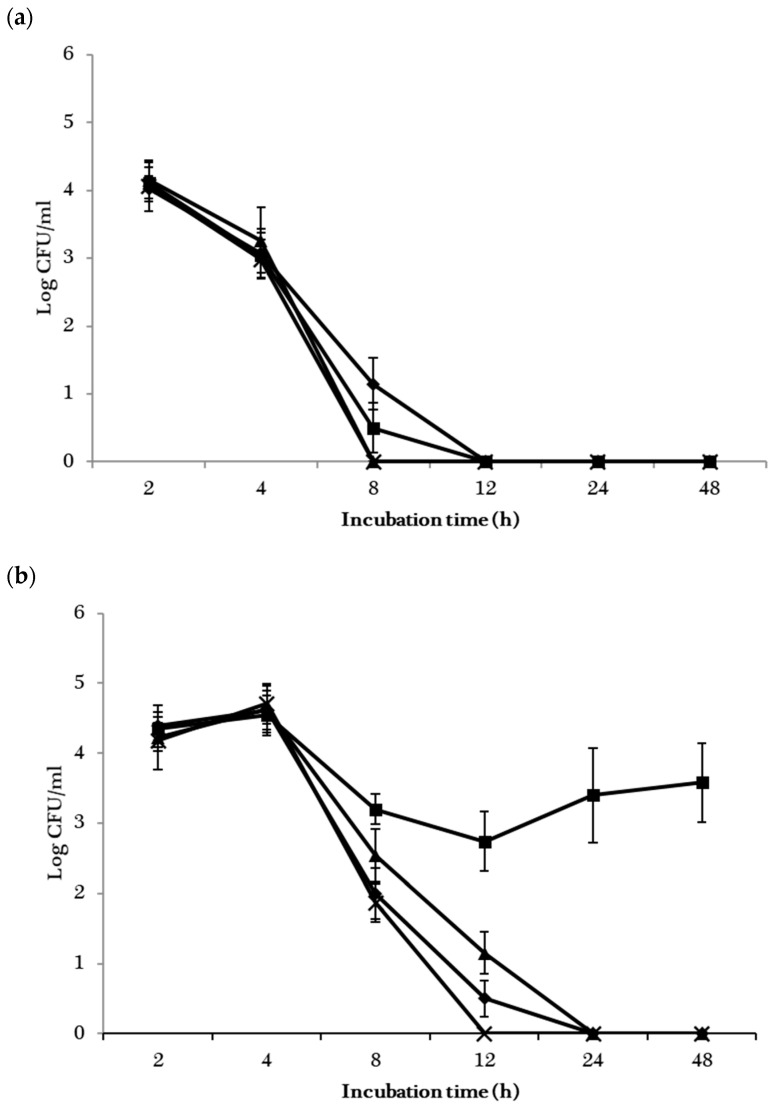
Mean populations of individual *S. enterica* (**a**) and *E. coli* (**b**) strains grown in PSB amended with cell-free supernatant of *L. rhamnousus* GG for 2, 4, 8, 12, 24, and 48 h. The strains included were *S*. Montevideo and *E. coli* F4546 (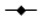), *S*. Stanley and *E. coli* K4492 (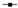), *S*. Cubana and *E. coli* H1730 (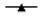), as well as *S*. Baildon and *E. coli* BAA-2326 (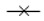).

The responses of the four individual *Salmonella* or EHEC strains to the CFS of *L. rhamnousus* GG over the 48-h incubation period are shown in Figure 4. The population of the four *Salmonella* strains decreased rapidly after 4 h of incubation, and populations of *S*. Baildon and *S*. Cubana fell below the detection limit of plate count assay after 8 h of incubation (Figure 4a). *S*. Montevideo and *S*. Stanley cells were not detectable after 12 h of incubation. The population of all 4 EHEC strains also decreased rapidly after the initial 4 h of incubation, and cells of *E. coli* BAA 2326 became undetectable after 12 h of incubation in PBS amended with CFS (Figure 4b). Cells of *E. coli* F4546 and H1730 were detectable till 24 h of incubation. However, the population of K4492 increased to 3.3 and 3.5 log CFU/mL at the 24 and 48 h sampling points after dropping from the 5.2 log CFU/mL inoculation level to 2.7 log CFU/mL during the initial 12 h of incubation.

## 4. Discussion

Results of the overall statistical analysis showed that co-cultures with *L. rhamnousus* GG had the lowest *Salmonella* and EHEC populations at both 25 and 37 °C compared to the co-cultures with other bacterial competitors (Table 2). Production of various organic acids and antimicrobial peptides including bacteriocin by *L. rhamnousus* GG might be the underlying mechanisms of the observed phenomenon. Organic acids can lower the pH of microbiological media and consequently inhibit the growth of bacterial pathogens [23]. Bacteriocins are proteinaceous or peptidic toxins produced by bacterial cells, which can kill susceptible pathogens by changing the permeability of bacterial membranes or interfering with the biological function of essential bacterial enzymes [24]. Distinct from the antimicrobial peptides produced by other *Lactobacillus* spp., those produced by *L. rhamnousus* GG belong to the least-characterized class of complex bacteriocin which had a broad spectrum of antagonistic activity against Gram-positive and Gram-negative organisms [24]. Previous studies have shown that the bacteriocin produced by *L. rhamnousus* GG can also inhibit the growth of some plant pathogens such as *Pseudomonas aeruginosa* and several major food spoilage microorganisms [25,26]. These findings suggest that the probiotic bacterium has a potential application as a biological control agent for improving the microbial safety of vegetable seeds, particularly sprout seeds. 

When *L. rhamnousus* GG was co-cultured with *Salmonella* or EHEC at 25 °C, the incubation time required for observable pathogen inhibition was relatively longer than incubation at 37 °C. Furthermore, *Salmonella* and EHEC population differences were much smaller between different sampling points at 25 °C (Figure 2). The relatively lower antagonistic ability of *L. rhamnousus* GG at 25 °C might be the result of a slower growth pace and accumulation of pathogen-cidal metabolites since this incubation temperature was less than optimal [23]. The growth rates of *L. rhamnousus* GG in MRS broth (pH 6.5) at 37 °C and 25 °C were reported as 0.95 and 0.36 OD600/h, respectively [27]. At 25 °C, the maximum *L. rhamnousus* GG population was 0.5–1.5 OD600 readings lower, and the time required for *L. rhamnousus* GG to reach the stationary phase was about 12 h longer than at 37 °C. When grown in UHT milk for 48 h, the amount of organic acids and acetaldehyde produced by *L. rhamnousus* GG at 30 °C was found significantly lower than at 37 °C [28]. Fayol-Messaoudi and coworkers [23] observed that when incubated at 32 °C, the killing activity of CFSs of the 24 h-culture of *L. rhamnousus* GG was significantly lower, although the lactic acid concentration in CFSs did not differ significantly from that at 37 °C. It is believed that the production of pathogen-cidal metabolites other than organic acid is also affected by the incubation temperature used to cultivate the probiotic bacterium. 

In general, lower (*p* < 0.05) populations of *Salmonella* than EHEC were observed in the co-cultures with *L. rhamnousus* GG (Table 2) or in PBS supplemented with its CFS (Figure 3). Similar observations were made by Arias and co-workers [29] who reported that the inhibition of *L. rhamnousus* and its CFS to *S*. Typhimurium was significantly stronger than to *E. coli* O157:H7. *E. coli* O157 strains are known to be more acid-resistant than *Salmonella* at pH from 2.5, 3.5, to 3.8 [30]. Higher tolerance of *E. coli* O157 to acetic acid and lactic acid were also reported [31,32]. Since the production of organic acid and the resulting low environmental pH are among the important antagonistic mechanisms utilized by *L. rhamnousus* GG [33], the ability of *E. coli* O157 to better adapt to the acidic environment might have contributed to the lower inhibitory effects of *L. rhamnousus* GG and its CFS observed in the present study. *E. coli* O104:H4 strain BAA 2326 used in the present study had a similar response to co-culture with *L. rhamnousus* GG and in PBS supplemented with its CFS compared to the two O157 strains, F4546, and H1730 (Figure 4b). The higher cell counts of *E. coli* K4492 in co-cultures with *L. rhamnousus* GG CFS indicates that the resistance of EHEC to the metabolites of *L. rhamnousus* GG is likely strain dependent.

Commercially used as a biocontrol agent, *P. fluorescens* A506 is known to compete with plant pathogen *E. amylovora* via preemptive utilization of growth-limiting nutrients at growth niches such as nactaries of pear blossoms [34]. In the present study, the use of a 100-fold higher start inoculum of *P. fluorescens* A506 over the tested *Salmonella* and EHEC strains resulted in a significant (*p* < 0.05), but overall a less than 1 log unit difference in *Salmonella* and EHEC population during the 72 h co-incubation period (Table 2, Table 3 and Table 4). The observed population reduction is likely the result of competition for nutrients by *P. fluorescens* A506 as the CFS of *P. fluorescens* A506 did not cause observable inhibition of *Salmonella* and EHEC (Figure 3). A similar observation has been noticed in several earlier studies. The presence of a *P. fluorescens* strain in the mixed culture with *E. coli* O157:H7 ATCC 43895 reportedly reduced the pathogen population overtime at 10, 15, and 25 °C [35]. The incubation temperature of 25 °C might also provide *P. fluorescens* A506 competition advantages over the two human pathogens, as the optimal growth temperature of *P. fluorescens* A506 is 25 °C while that of *Salmonella* and EHEC is 37 °C. 

Slight, but significant (*p* < 0.05) differences in *Salmonella* and EHEC populations were observed when they were co-cultured with *B. mojavensis* RRC101 and *B. subtilis* ATCC6051 compared to the pathogen populations in the control cultures (Table 2). The antagonistic activities of the two biocontrol agents were reported to rely on the release of specific fungicidal cyclic lipopeptides and/or bio-surfactants [36,37]. Whether the bio-surfactants can effectively inhibit the growth of *Salmonella* and EHEC remains contradictory. The surfactin extracted from the 7-day culture of *B. licheniformis* M104 inhibited the growth of *S*. Typhimurium ATCC 14028 and two *E. coli* strains ATCC 11775 and 11246 in a disc diffusion assay [38]. However, Mireles and coworkers found that surfactins of *B. subtilis* could only inhibit biofilm formation, but not the growth of *S. enterica* and *E. coli* cells [39]. The slight reductions in *Salmonella* and EHEC populations resulted from the co-presence of *B. mojavensis* RRC101 and *B. subtilis* ATCC 6051 in the present study are likely the result of another mechanism such as the competition for nutrients in microbiological media. 

Using multiple biocontrol agents simultaneously could be one of the solutions to the unsatisfactory inhibition to target pathogen(s) by individual biocontrol agents. Knowing the inhibitory mechanisms of each biocontrol agent is crucial in finding the most efficient components of a biological control cocktail, ideally, different modes of action can be applied simultaneously to avoid possible adaption of the target pathogen(s) [40]. It is also important to understand if synergistic/antagonistic effects exist among all candidates used in the same cocktail. Ye and coworkers [16] previously reported the success in using a combination of plant bacterium *E. asburiae* JX1 and a cocktail of *Salmonella* lytic phages to achieve a 3-log unit reduction in the *Salmonella* population on sprouting mung bean and alfalfa seeds. Other studies also described the potential of using cocktails of antagonistic strains in the control of various plant and human pathogens [40,41,42]. In the current study, slightly lower pathogen populations were observed when the two cocktails were used (Table 2). It is not yet clear if the slightly larger pathogen population differences caused by the presence of the two cocktails were the results of synergetic interactions among the biocontrol agents or simply the effect of a greater overlap of nutrition requirements among cells of multiple biocontrol agents and *Salmonella*/EHEC strains. Further research is needed to fully understand and improve the inhibitory effects of the cocktails on human pathogens in the co-culture systems. 

Although the presence of *L. rhamnousus* GG in a co-culture had the lowest *Salmonella* and EHEC populations, adding *L. rhamnousus* GG to Cocktail 1 did not significantly (*p* > 0.05) change the ability of the biocontrol cocktail in inhibiting the growth of *Salmonella* (Table 2). The reduction in the EHEC population caused by the presence of the two cocktails differed only by 0.1 log units. It was found in the study that the pH of 72-h mixed cultures containing Cocktail 2 and *Salmonella* or EHEC ranged from 6.8 to 7.2, which was higher than the pH of 3.7–4.3 from the co-cultures of *L. rhamnousus* GG with *Salmonella*/EHEC (data not shown). This indicates a poor *L. rhamnousus* GG growth in the mixed culture containing Cocktail 2, subsequently a low level of accumulation of anti-*Salmonella* and –EHEC metabolites in the cocktail. Since the biocontrol agents used in Cocktail 2 had an optimal growth temperature of 25 °C, *L. rhamnousus* GG was likely to be outgrown by the biocontrol agents in the cocktail at this incubation temperature. The relatively higher pH in Cocktail 2 could also affect the function of non-organic acid antimicrobial molecules produced by *L. rhamnousus* GG. It has been reported that the antagonistic compounds produced by *L. rhamnousus* GG suppressed the growth of *S*. Typhimurium at pH 4.5 but not at pH 6.5 [23]. Synergistic effects between lactic acid and non-lactic acid antimicrobial molecules released by *Lactobacillus* strains have been described by Alakomi and coworkers [43] who reported that the lactic acid molecules can modify the permeability of the outer membrane of gram-negative pathogens, allowing antimicrobial molecules to more easily across cell membranes of the target bacteria. As stated previously, a good biocontrol cocktail should not contain bacterial strains which are antagonistic with one another. 

Although not as competitive as *L. rhamnousus* GG in inhibiting the growth of *Salmonella* and EHEC, the three biocontrol agents significantly (*p* < 0.05) reduced the attachment of *Salmonella* and EHEC cells to tested vegetable seeds (Table 5). The lower level of *Salmonella* and EHEC attachment caused by the co-presence of these bacterial strains might partially result from the competition among cells of participating bacterial strains including those of human pathogens for limited niches of attachment on vegetable seeds. Ideal attachment niches for microorganisms usually include the wrinkles, cracks, and crevices on the surface of vegetable seeds [44]. The production of surfactin-like molecules by *L. rhamnousus* GG [45] and the two *Bacillus* strains [37] might have interfered with the attachment by *Salmonella* and *E. coli* cells to seed surfaces. Most significant reductions in *Salmonella* and EHEC attachment to vegetable seeds were observed when *P. fluorescens* A506 was present. The reason for the observed phenomenon is unclear, but it may because *P. fluorescens* is a better colonizer that can attach firmly to seeds, roots, and various plant tissues in the presence of many other competitive plant microorganisms [46]. 

The interactions between plant and human pathogens have been discussed widely as plant pathogens might play a role in the survival of human pathogens within various plant hosts [47,48]. Certain activities of plant pathogens (e.g., breach of plant cell walls, the necrotic release of nutrients from plant cells and suppression of plant immunity, etc.) might boost the survival of human pathogens that are present in nearby proximity. Esseilli and coworkers [49] reported that the necrotic lesions created by *Xanthomonas campestris* pv. *vitians* or cucumber mosaic virus strain Fny enhanced the postharvest survival of human pathogens on leafy green vegetables. Water-soaking of tomato leaves from the infection by *X. euvesicatoria* and *X. gardneri* supported the persistence and/or growth of *S. enterica* [50]. The majority of the known interactions between plant and human pathogens were observed within mature plant hosts. Although *P. syringae* and its multiple pathovars (e.g., syringae, tomato) have been reported to enhance the survival and growth of *S*. Typhimurium and pathogenic *E. coli* on leaves of different plants [51], Pst DC3000 in the current study only slightly inhibited the growth of the two pathogens (Table 2). Whether and how the two types of pathogens interact with each other outside mature plants, such as on vegetable seeds and seedlings, demands further, and more systematic investigation. 

## 5. Conclusions

Biological control of human pathogens on vegetable seeds at both pre- and post-harvest stages has drawn the attention of many researchers, as it can lead to the production of vegetable seeds and fresh produce with high microbial qualities. In this study, significantly lower populations of *Salmonella* and EHEC were observed when they were in co-cultures with *L. rhamnousus* GG in M/T broth and PBS supplemented with *L. rhamnousus* GG CFS. Since *L. rhamnousus* GG is a probiotic organism and its beneficial effect on human gut health has been proven in numerous clinical trials, it could be a potential biocontrol agent to improve the microbial safety of vegetable seeds, especially at postharvest stages. Although not as competitive as *L. rhamnousus* GG in inhibiting the growth of *Salmonella* and EHEC, the three biocontrol agents were more effective in competing with *Salmonella* and EHEC for attachment to vegetable seeds. Thus, in addition to the control of plant pathogens, these agents could also be used to control human pathogens during vegetable seed production. The different responses of the *S. enterica* and EHEC strains to each competitive bacterium and cocktails highlight the importance of choosing appropriate biocontrol agent(s) for targeted pathogens. Future studies should focus on the identification of additional effective competitive bacterial strains with negligible adverse influences on seed/fresh produce quality as well as a better understanding of the synergetic mechanisms among participating bacterial strains to maximize their antagonistic effects. The effectiveness of the identified biocontrol agents should be verified during pilot trials of vegetable seed production and storage after laboratory evaluations. 

## Figures and Tables

**Table 1:** Bacterial strains used in this study.

Nalidixic Acid Resistant Derivate	Serovar/Strain	Source
*Salmonella enterica*	Stanley	Sprout-related outbreak, Finland, Canada, and the US, 1997
	Baildon	Tomato- and lettuce-related outbreaks, the US, 1999
	Montevideo	Tomato-related outbreak, the US, 1993
	Cubana	Sprout-related outbreak, the US, 2012
Enterohemorrhagic *Escherichia coli*	F4546	Sprout-related outbreak, the US, 1997
	BAA-2326	Fenugreek sprout-related outbreak, Germany, 2011
	K4492	Spinach-related outbreak, the US, 2006
	H1730	Lettuce-related outbreak, the US, 2003
*Pseudomonas fluorescenes*	A506	Commercial biocontrol agent for *Erwinia amylovora* (fire blight) on fruits
*Bacillus mojavensis*	RRC 101	Commercial biocontrol agent for *Fusarium verticillioides* in maize and other crops; surfactin-producing
*Bacillus subtilis*	ATCC 6051	surfactin-producing strain
*Lactobacillus rhamnosus*	GG	Commercial probiotic
*Pseudomonas syringae* pv. *tomato*	DC 3000	Frequent seed-borne pathogen on tomato plant

**Table 2:** Overall mean *S. enterica* and EHEC populations in co-cultures with different competitive bacterial strains and at different sampling points.

Main Effect	Mean Population of *Salmonella* or EHEC (log CFU/mL) as Influenced by		Main Effect	Mean Population (log CFU/mL) of *Salmonella* and EHEC as Influenced by
	*S. enterica*^1^ (*n* = 540)	*E. coli*^2^ (*n* = 540)		
**Competitive bacteria presence**			***S. enterica* strains used**	
Control (*n* = 60)	7.8 ± 0.26 A	8.1 ± 0.26 A	*S*. Montevideo (*n* = 135)	6.8 ± 0.19 A
*P. syringae* pv. *tomato* DC 3000 (*n* = 60)	7.4 ± 0.24 B	7.8 ± 0.26 B	*S*. Baildon (*n* = 135)	6.6 ± 0.19 A
*B. subtilis* ATCC 6051 (*n* = 60)	7.3 ± 0.22 BC	7.6 ± 0.25 C	*S*. Stanley (*n* = 135)	6.1 ± 0.20 B
*B. mojavensis* RRC 101 (*n* = 60)	7.2 ± 0.21 C	7.5 ± 0.24 C	*S*. Cubana (*n* = 135)	6.1 ± 0.19 B
*P. fluorescenes* A506 (*n* = 60)	7.0 ± 0.22 D	7.4 ± 0.24 D	***E. coli* strains used**	
Cocktail 1 ^3^ (*n* = 60)	6.9 ± 0.24 D	7.3 ± 0.24 D	*E. coli* H1730 (*n* = 135)	7.9 ± 0.12 A
Cocktail 2 ^4^ (*n* = 60)	6.9 ± 0.24 D	7.2 ± 0.24 E	*E. coli* K4492 (*n* =135)	7.7 ± 0.13 A
*L. rhamnosus* GG (25 °C; *n* = 60)	6.0 ± 0.23 E	6.8 ± 0.22 F	*E. coli* F4546 (*n* = 135)	7.1 ± 0.17 B
*L. rhamnosus* GG (37 °C; *n* = 60)	2.5 ± 0.31 F	6.6 ± 0.15 G	*E. coli* BAA 2326 (*n* = 135)	6.4 ± 0.17 C
**Sampling points (h) used**				
24 (*n* = 108)	7.5 ± 0.15 A	8.7 ± 0.07 B		
48 (*n* = 108)	7.5 ± 0.23 A	9.0 ± 0.06 A		
72 (*n* = 108)	6.9 ± 0.26 B	8.4 ± 0.10 C		
12 (*n* = 108)	6.2 ± 0.12 C	6.3 ± 0.14 D		
6 (*n* = 108)	3.8 ± 0.24 D	4.1 ± 0.11 E		

^1^ Mean populations of *S. enterica* within the same variable in a column not followed by the same letter are significantly different (*p* < 0.05). ^2^ Mean populations of *E. coli* within the same comparable in a column not followed by the same letter are significantly different (*p* < 0.05). ^3^ A mixed culture of *P. fluorescenes* AS06, *B. mojavensis* RRC 101 and *B. subtilis* ATCC 6051. ^4^ A mixed culture of *P. fluorescenes* AS06, *B. mojavensis* RRC 101, *B. subtilis* ATCC 6051 and *L. rhamnosus* GG.

**Table 3:** Mean populations of individual *Salmonella* strains in co-cultures with different competitive bacterial strains and the plant pathogen at 25 °C.

*Salmonella Strains*	Mean Population of *Salmonella* ^1^ (log CFU/mL) (*n* = 480)							
	*P. fluorescens* A506	Cocktail 2	Cocktail 1	*L. rhamnosus* GG	Pst DC3000	*B. mojavensis* RRC101	Control	*B. subtilis* ATCC6051
Montevideo (*n* = 15)	7.6 ± 0.39 aAB	7.4 ± 0.44 aAB	7.3 ± 0.44aAB	6.5 ± 0.39 aB	7.8 ± 0.45 aA	7.6 ± 0.40 aAB	8.0 ± 0.55 aA	7.6 ± 0.41 aAB
Baildon (*n* = 15)	7.2 ± 0.45 aA	7.2 ± 0.46 aA	7.4 ± 0.46 aA	5.9 ± 0.53 aB	7.8 ± 0.41 aA	7.4 ± 0.39 aA	8.0 ± 0.49 aA	7.6 ± 0.38 aA
Stanley (*n* = 15)	6.8 ± 0.47 aAB	6.3 ± 0.46 aAB	6.5 ± 0.48aAB	5.7 ± 0.47 aB	7.2 ± 0.46 aA	7.0 ± 0.45 aAB	7.5 ± 0.56 aA	7.0 ± 0.49 aAB
Cubana (*n* = 15)	6.5 ± 0.46 aAB	6.5 ± 0.54 aAB	6.5 ± 0.45 aAB	5.9 ± 0.49 aB	6.8 ± 0.54 aAB	6.8 ± 0.45 aAB	7.9 ± 0.50 aA	6.8 ± 0.47 aAB

^1^ Mean values within a column not followed by the same lowercase letter are significantly different (*p* < 0.05). Mean values within a row not followed by the same uppercase letter are significantly different (*p* < 0.05).

**Table 4:** Mean populations of individual EHEC strains in co-cultures with different competitive bacterial strains and the plant pathogen at 25 °C.

EHEC Strains	Mean Population of EHEC ^1^ (log CFU/mL) (*n* = 480)							
	*P. fluorescens* A506	Cocktail 2	Cocktail 1	*L. rhamnosus* GG	Pst DC3000	*B. mojavensis* RRC101	Control	*B. subtilis* ATCC6051
H1730 (*n* = 15)	8.2 ± 0.30 aAB	7.5 ± 0.35 abAB	7.7 ± 0.35 abAB	7.4 ± 0.38 aB	8.6 ± 0.33 aAB	8.2 ± 0.33 aAB	8.6 ± 0.42 abA	8.3 ± 0.32 aAB
K4492 (*n* = 15)	8.1 ± 0.40 aAB	7.6 ± 0.43 aAB	7.7 ± 0.42 aAB	7.1 ± 0.46 aB	8.4 ± 0.38 aA	8.3 ± 0.38 aA	8.7 ± 0.41 aA	8.3 ± 0.39 aA
F4546 (*n* = 15)	6.9 ± 0.56 abA	7.3 ± 0.58 abA	7.3 ± 0.56 abA	6.7 ± 0.47 aA	7.7 ± 0.52 abA	7.3 ± 0.53 abA	7.9 ± 0.52 abA	7.2 ± 0.54 abA
BAA2326 (*n* = 15)	6.3 ± 0.48 bA	6.2 ± 0.53 bA	6.6 ± 0.49 bA	6.0 ± 0.43 aA	6.6 ± 0.60 bA	6.7 ± 0.57 bA	7.3 ± 0.65 bA	6.6 ± 0.59 bA

^1^ Mean values within a column not followed by the same lowercase letter are significantly different (*p* < 0.05). Mean values within a row not followed by the same uppercase letter are significantly different (*p* < 0.05).

**Table 5:** Mean percentages of attached cells of the four-strain *S. enterica* or EHEC mixtures as affected by individual competitive bacterial strains and their cocktail.

	Percentage (%) of Attached Cells	
	*Salmonella*^1^ (*n* = 84)	EHEC (*n* = 84)
***As influenced by competitive strains***		
Control (*n* = 12)	10.5 ± 0.38 A	3.9 ± 0.14 A
*L. rhamnosus* GG (*n* = 12)	9.4 ± 0.33 B	3.4 ± 0.11 B
Cocktail 2 ^2^ (*n* = 12)	9.0 ± 0.29 C	3.1 ± 0.18 C
*B*. *mojavensis* RRC 101 (*n* = 12)	7.9 ± 0.21 D	2.9 ± 0.16 C
*B*. *subtilis* ATCC 6051 (*n* = 12)	8.1 ± 0.24 D	2.8 ± 0.23 C
*P*. *syringae* pv. *tomato* DC 3000 (*n* = 12)	8.2 ± 0.27 D	3.5 ± 0.20 B
*P*. *fluorescenes* A506 (*n* = 12)	7.0 ± 0.20 E	2.4 ± 0.12 D
***On different seed types***		
Fenugreek (*n* = 21)	12.5 ± 0.19 A	6.7 ± 0.11 A
Alfalfa (*n* = 21)	11.8 ± 0.08 B	2.0 ± 0.08 B
Lettuce (*n* = 21)	8.9 ± 0.08 C	1.7 ± 0.04 C
Tomato (*n* = 21)	ND ^3^	ND

^1^ Mean percentages of attachment within a column not followed by the same letter are significantly different (*p* < 0.05). ^2^ Cocktail 2: A mixed culture of *P. fluorescenes* AS06, *B. mojavensis* RRC 101, *B. subtilis* ATCC 6051, and *L. rhamnosus* GG. ^3^ Attachment not detected.

## Data Availability

The datasets generated for this study are available on request to the corresponding author.

## References

[1] Como-Sabetti K., Reagan S., Allaire S., Parrott K., Simonds C., Hrabowy S., Ritter B., Hall W., Altamirano J., Martin R. (1997). Outbreaks of *Escherichia coli* O157:H7 infection associated with eating alfalfa sprouts—Michigan and Virginia, June–July 1997. Morb. Mortal. Wkly. Rep..

[2] Mahon B.E., Pönkä A., Hall W.N., Komatsu K., Dietrich S.E., Siitonen A., Cage G., Hayes P.S., Lambert-Fair M.A., Bean N.H. (1997). An international outbreak of *Salmonella* infections caused by alfalfa sprouts grown from contaminated seeds. J. Infect. Dis..

[3] Winthrop K., Palumbo M., Farrar J., Mohle-Boetani J., Abbott S., Beatty M., Inami G., Werner S. (2003). Alfalfa sprouts and *Salmonella* Kottbus infection: A multistate outbreak following inadequate seed disinfection with heat and chlorine. J. Food Prot..

[4] Taormina P.J., Beuchat L.R., Slutsker L. (1999). Infections associated with eating seed sprouts: An international concern. Emerg. Inf. Dis..

[5] Howard M.B., Hutcheson S.W. (2003). Growth dynamics of *Salmonella enterica* strains on alfalfa sprouts and in waste seed irrigation water. Appl. Environ. Microbiol..

[6] Deering A.J., Jack D.R., Pruitt R.E., Mauer L.J. (2015). Movement of *Salmonella* serovar Typhimurium and *E. coli* O157:H7 to ripe tomato fruit following various routes of contamination. Microorganisms.

[7] Andrews W., Hammack T., Amaguana R. (2007). Chapter 5, Salmonella. In Bacteriological Analytical Manual. US Food and Drug Administration. http://www.cfsan.fda.gov/ebam/bam-5.htm.

[8] Feng P., Weagant S., Jinneman K. Bacteriological Analytical Manual, Chapter 4A, Diarrheagenic *Escherichia coli*. http://www.fda.gov/Food/FoodScienceResearch/LaboratoryMethods/ucm070080.htm.

[9] Fett W. (2002). Factors affecting the efficacy of chlorine against *Escherichia coli* O157:H7 and *Salmonella* on alfalfa seed. Food Microbiol..

[10] Cooley M.B., Miller W.G., Mandrell R.E. (2003). Colonization of *Arabidopsis thaliana* with *Salmonella enterica* and enterohemorrhagic *Escherichia coli* O157:H7 and competition by *Enterobacter asburiae*. Appl. Environ. Microbiol..

[11] Davidson P.M., Harrison M.A. (2002). Resistance and adaptation to food antimicrobials, sanitizers, and other process controls. Food Technol..

[12] Kamilova F., Kravchenko L.V., Shaposhnikov A.I., Makarova N., Lugtenberg B. (2006). Effects of the tomato pathogen *Fusarium oxysporum* f. sp. radicis-lycopersici and of the biocontrol bacterium Pseudomonas fluorescens WCS365 on the composition of organic acids and sugars in tomato root exudate. Mol. Plant-Microbe Interact..

[13] Kamilova F., Validov S., Azarova T., Mulders I., Lugtenberg B. (2005). Enrichment for enhanced competitive plant root tip colonizers selects for a new class of biocontrol bacteria. Environ. Microbiol..

[14] Fessehaie A., Walcott R. (2005). Biological control to protect watermelon blossoms and seed from infection by *Acidovorax avenae* subsp. citrulli. Phytopathology.

[15] Liao C.H. (2008). Growth of *Salmonella* on sprouting alfalfa seeds as affected by the inoculum size, native microbial load and *Pseudomonas fluorescens* 2–79. Lett. Appl. Microbiol..

[16] Ye J., Kostrzynska M., Dunfield K., Warriner K. (2010). Control of *Salmonella* on sprouting mung bean and alfalfa seeds by using a biocontrol preparation based on antagonistic bacteria and lytic bacteriophages. J. Food Prot..

[17] Compant S., Duffy B., Nowak J., Clément C., Barka E.A. (2005). Use of plant growth-promoting bacteria for biocontrol of plant diseases: Principles, mechanisms of action, and future prospects. Appl. Environ. Microbiol..

[18] Cabrefiga J., Bonaterra A., Montesinos E. (2007). Mechanisms of antagonism of *Pseudomonas fluorescens* EPS62e against *Erwinia amylovora*, the causal agent of fire blight. Int. Microbiol..

[19] Bredholt S., Nesbakken T., Holck A. (2001). Industrial application of an antilisterial strain of *Lactobacillus sakei* as a protective culture and its effect on the sensory acceptability of cooked, sliced, vacuum-packaged meats. Int. J. Food Microbiol..

[20] Nitschke M., Araújo L., Costa S., Pires R., Zeraik A., Fernandes A., Freire D., Contiero J. (2009). Surfactin reduces the adhesion of food-borne pathogenic bacteria to solid surfaces. Lett. Appl. Microbiol..

[21] De Keersmaecker S.C., Verhoeven T.L., Desair J., Marchal K., Vanderleyden J., Nagy I. (2006). Strong antimicrobial activity of *Lactobacillus rhamnosus* GG against *Salmonella typhimurium* is due to accumulation of lactic acid. FEMS Microbiol. Lett..

[22] Cui Y., Walcott R., Chen J. (2017). Differential attachment of *Salmonella enterica* and enterohemorrhagic *Escherichia coli* to alfalfa, fenugreek, lettuce, and tomato seeds. Appl. Environ. Microbiol..

[23] Fayol-Messaoudi D., Berger C.N., Coconnier-Polter M.-H., Lievin-Le Moal V., Servin A.L. (2005). pH-, lactic acid-, and non-lactic acid-dependent activities of probiotic lactobacilli against *Salmonella enterica* serovar Typhimurium. Appl. Environ. Microbiol..

[24] Pithva S., Ambalam P., Dave J., Vyas B. (2011). Antimicrobial Peptides of Probiotic *Lactobacillus* Strains. https://www.researchgate.net/publication/258338154_Antimicrobial_Peptides_of_Probiotic_Lactobacillus_strains.

[25] Alexandre Y., Le Berre R., Barbier G., Le Blay G. (2014). Screening of *Lactobacillus* spp. for the prevention of *Pseudomonas aeruginosa* pulmonary infections. BMC Microbiol..

[26] Tharmaraj N., Shah N.P. (2009). Antimicrobial effects of probiotics against selected pathogenic and spoilage bacteria in cheese-based dips. Int. Food. Res. J..

[27] Deepika G., Karunakaran E., Hurley C.R., Biggs C.A., Charalampopoulos D. (2012). Influence of fermentation conditions on the surface properties and adhesion of *Lactobacillus rhamnosus* GG. Microb. Cell Factories.

[28] Østlie H.M., Treimo J., Narvhus J.A. (2005). Effect of temperature on growth and metabolism of probiotic bacteria in milk. Int. Dairy J..

[29] Arias O.A., Reyes M.M., Navarro V.M.L., Solis C.Y., Márquez G.M., Sanchez S.G., Snell C.R., Zuñiga R.R. Antagonistic Effect of Probiotic Strains against Two Pathogens: *Salmonella* Typhimurium and *E. coli* O157:H7 Resistant to Antibiotics. https://www.redalyc.org/pdf/730/73029399005.pdf.

[30] Breidt F., Kay K., Cook J., Osborne J., Ingham B., Arritt F. (2013). Determination of 5-log reduction times for *Escherichia coli* O157:H7, *Salmonella enterica*, or *Listeria monocytogenes* in acidified foods with pH 3.5 or 3.8. J. Food Prot..

[31] Berry E.D., Cutter C.N. (2000). Effects of acid adaptation of *Escherichia coli* O157:H7 on efficacy of acetic acid spray washes to decontaminate beef carcass tissue. Appl. Environ. Microbiol..

[32] Brackett R., Hao Y.-Y., Doyle M. (1994). Ineffectiveness of hot acid sprays to decontaminate *Escherichia coli* O157:H7 on beef. J. Food Prot..

[33] Vanderhoof J.A., Whitney D.B., Antonson D.L., Hanner T.L., Lupo J.V., Young R.J. (1999). *Lactobacillus* GG in the prevention of antibiotic-associated diarrhea in children. J. Pediatr..

[34] Stockwell V., Johnson K., Sugar D., Loper J. (2010). Control of fire blight by *Pseudomonas fluorescens* A506 and *Pantoea vagans* C9-1 applied as single strains and mixed inocula. Phytopathology.

[35] Samelis J., Sofos J.N. (2002). Role of glucose in enhancing the temperature-dependent growth inhibition of *Escherichia coli* O157:H7 ATCC 43895 by a *Pseudomonas* sp. Appl. Environ. Microbiol..

[36] Bacon C.W., Hinton D.M. (2002). Endophytic and biological control potential of *Bacillus mojavensis* and related species. Biol. Control.

[37] Snook M.E., Mitchell T., Hinton D.M., Bacon C.W. (2009). Isolation and characterization of Leu7-surfactin from the endophytic bacterium *Bacillus mojavensis* RRC 101, a biocontrol agent for *Fusarium verticillioides*. J. Agric. Food Chem..

[38] Gomaa E.Z. (2013). Antimicrobial activity of a biosurfactant produced by *Bacillus licheniformis* strain M104 grown on whey. Braz. Arch. Biol. Technol..

[39] Mireles J.R., Toguchi A., Harshey R.M. (2001). *Salmonella enterica* serovar Typhimurium swarming mutants with altered biofilm-forming abilities: Surfactin inhibits biofilm formation. J. Bacteriol..

[40] Vijayakumar P.P., Muriana P.M. (2017). Inhibition of *Listeria monocytogenes* on ready-to-eat meats using bacteriocin mixtures based on mode-of-action. Foods.

[41] Bonaterra A., Badosa E., Cabrefiga J., Francés J., Montesinos E. (2012). Prospects and limitations of microbial pesticides for control of bacterial and fungal pomefruit tree diseases. Trees.

[42] Nisbet D. (2002). Defined competitive exclusion cultures in the prevention of enteropathogen colonisation in poultry and swine. Antonie Leeuwenhoek.

[43] Alakomi H.-L., Skyttä E., Saarela M., Mattila-Sandholm T., Latva-Kala K., Helander I. (2000). Lactic acid permeabilizes gram-negative bacteria by disrupting the outer membrane. Appl. Environ. Microbiol..

[44] Charkowski A.O., Sarreal C.Z., Mandrell R.E. (2001). Wrinkled alfalfa seeds harbor more aerobic bacteria and are more difficult to sanitize than smooth seeds. J. Food Prot..

[45] Mujumdar S., Bashetti S., Pardeshi S., Thombre R.S. (2016). Industrial applications of biosurfactants. Industrial Biotechnology: Sustainable Production and Bioresource Utilization.

[46] Dekkers L.C., Bloemendaal C.J.P., de Weger L.A., Wijffelman C.A., Spaink H.P., Lugtenberg B.J. (1998). A two-component system plays an important role in the root-colonizing ability of *Pseudomonas fluorescens* strain WCS365. Mol. Plant-Microbe Interact..

[47] Aruscavage D., Miller S.A., Lewis Ivey M.L., Lee K., LeJeune J.T. (2008). Survival and dissemination of *Escherichia coli* O157:H7 on physically and biologically damaged lettuce plants. J. Food Prot..

[48] Cooley M.B., Chao D., Mandrell R.E. (2006). *Escherichia coli* O157:H7 survival and growth on lettuce is altered by the presence of epiphytic bacteria. J. Food Prot..

[49] Esseili M.A., Chin A., Saif L., Miller S.A., Qu F., Lewis Ivey M.L., Wang Q. (2015). Postharvest survival of porcine sapovirus, a human norovirus surrogate, on phytopathogen-infected leafy greens. J. Food Prot..

[50] Potnis N., Soto-Arias J.P., Cowles K.N., van Bruggen A.H., Jones J.B., Barak J.D. (2014). *Xanthomonas perforans* colonization influences *Salmonella enterica* in the tomato phyllosphere. Appl. Environ. Microbiol..

[51] Poza-Carrion C., Suslow T., Lindow S. (2013). Resident bacteria on leaves enhance survival of immigrant cells of *Salmonella enterica*. Phytopathology.

